# POLYPHARMACY IN DIABETIC PEOPLE: EVIDENCE FROM THE ENGLISH LONGITUDINAL STUDY OF AGEING (ELSA)

**DOI:** 10.1093/geroni/igz038.1794

**Published:** 2019-11-08

**Authors:** Yun-Ting Huang, Paola Zaninotto, Andrew Steptoe, Li Wei

**Affiliations:** University College London, London, United Kingdom

## Abstract

Diabetes among older people is becoming more common worldwide, and usually accompanied by polypharmacy. However, the role of polypharmacy in older people with diabetes remains uncertain. A nationally representative cross-sectional study, ELSA 2012/2013, was used and 7729 participants aged 50-109 were investigated. Polypharmacy was defined as taking five to nine long-term used medications daily for chronic diseases or chronic symptoms, while using ten or more medications was excessive polypharmacy. The presence of illness was defined as either self-reported diagnosis or being prescribed specific medications for the condition. Data showed the prevalence of polypharmacy was 21.4%, and only 3% was excessive polypharmacy. 51.6% of diabetic people reported polypharmacy and 10.2% excessive polypharmacy. These rates were significantly higher than the 16.4% polypharmacy and 1.8% excessive polypharmacy among people without diabetes (p < 0.001). Among people with three or more comorbidities, polypharmacy was present in 61.5% of people with diabetes, compared with 36.0% in people without diabetes. Significant risk factors for polypharmacy were diabetes (Relative-risk ratios/RRR=4.06, 95% CI 3.38, 4.86), older age (RRR=1.02, 95% CI 1.01, 1.03), male (RRR=0.64, 95% CI 0.55, 0.75), more comorbidity (RRR=2.46, 95% CI 2.30, 2.62), living with a partner (RRR=1.20, 95% CI 1.01, 1.42), and less wealth (RRR=0.93, 95% CI 0.87, 0.98). However, age, cohabitation, and wealth were not significantly related to excessive polypharmacy. Diabetes and the number of comorbidities were predominant risk factors for excessive polypharmacy. Current evidences confirmed both health condition and socioeconomic status were associated with medication use in older adults.

